# Inhibition of calpain delays early muscle atrophy after rotator cuff tendon release in sheep

**DOI:** 10.14814/phy2.13833

**Published:** 2018-11-04

**Authors:** Severin Ruoss, Philipp Kindt, Linus Oberholzer, Marco Rohner, Ladina Jungck, Sara Abdel‐Aziz, Daniel Fitze, Andrea B. Rosskopf, Karina Klein, Brigitte von Rechenberg, Christian Gerber, Karl Wieser, Martin Flück

**Affiliations:** ^1^ Laboratory for Muscle Plasticity University of Zurich Zurich Switzerland; ^2^ Vetsuisse Faculty University of Zurich Zurich Switzerland; ^3^ Faculty of Medicine University of Zurich Zurich Switzerland; ^4^ Radiology Department Balgrist University Hospital University of Zurich Zurich Switzerland; ^5^ Competence Center for Applied Biotechnology and Molecular Medicine (CABMM) University of Zurich Zurich Switzerland; ^6^ Department of Orthopaedics Balgrist University Hospital University of Zurich Zurich Switzerland

**Keywords:** Atrophy, calpain, costamere, rotator cuff tear, tenotomy

## Abstract

Chronic rotator cuff (RC) tears are characterized by retraction, fat accumulation, and atrophy of the affected muscle. These features pose an intractable problem for surgical repair and subsequent recovery, and their prevention may be easier than reversal. Using an established ovine model, we tested the hypothesis that inhibition of the protease calpain mitigates *m. infraspinatus* atrophy by preservation of the myofibers’ structural anchors in the sarcolemma (the costameres). Already 2 weeks of distal tendon release led to a reduction in muscle volume (−11.6 ± 9.1 cm^3^, *P *=* *0.038) and a 8.3% slow‐to‐fast shift of the fiber area (*P *=* *0.046), which were both entirely abolished by chronic local administration of the calpain inhibitor calpeptin alone, and in combination with sildenafil. Calpain inhibition blunted the retraction of the muscle‐tendon unit by 0.8–1.0 cm (*P *=* *0.020) compared with the control group, and prevented cleavage of the costameric protein talin. Calpain 1 and 2 protein levels increased in the medicated groups after 4 weeks, counteracting the efficacy of calpeptin. Hence atrophic changes emerged after 4 weeks despite ongoing treatment. These findings suggest that the early muscular adaptations in the specific case of RC tear in the ovine model are indistinguishable from the atrophy and slow‐to‐fast fiber transformation observed with conventional unloading and can be prevented for 2 weeks. Concluding, calpain is a potential target to extend the temporal window for reconstruction of the ruptured RC tendon before recovery turns impossible.

## Introduction

Chronic rotator cuff (RC) tendon tears are among the most frequent causes of shoulder dysfunction (Milgrom et al. 1995; Sher et al. 1995) leading to various functional deficits including weakness and limited range of motion (Duckworth et al. 1999). These tears often occur unnoticed and therefore remain untreated for months or years until they create functional impairment or pain (Milgrom et al. 1995; Yamaguchi et al. 2006). At this point, the affected RC muscle is already shortened, contractile tissue is lost and muscle volume predominantly consists of fat and connective tissue; a transformation that substantially complicates treatment or even renders it impossible (Bartolozzi et al. 1994; Goutallier et al. 1994; Gladstone et al. 2007). A time line of the progression of atrophy and degeneration in man is essentially unavailable and researchers are prompted to use animal models to characterize muscle loss in chronic RC tear. The ovine model is considered valid as relevant parameters like fiber type composition (Suzuki 1995; Lovering and Russ 2008) or changes in fat fraction (Pfirrmann et al. 2004; Ruoss et al. 2017) are comparable to changes observed in human RC muscles, as opposed to small animal models, which do not present these features (Lebaschi et al. 2016; Rui et al. 2016). Studies in sheep have shown that muscle atrophy after RC tear is already present after 2 weeks and once a chronic transformation is fully established (i.e., atrophy and intramuscular fat content compare to chronic human RC tear), reversal fails despite gradual mechanical reloading and/or anabolic steroid treatment (Gerber et al. 2004, 2012; Ruoss et al. 2017). Thus, successful prevention of early adaptations to tear‐induced unloading could extend the window of opportunity for successful surgical repair and recovery of muscle function. Early adaptation to unloading is characterized by decreasing muscle volume with little to no fat accumulation (Rowshan et al. 2010; Uhthoff et al. 2014; Ruoss et al. 2017). Loss of contractile tissue leads to both radial and longitudinal atrophy, the latter leading to the clinical phenomenon of “myotendinous retraction” which is related to serial removal of sarcomeres from myofibrils (Jamali et al. 2000; Ward et al. 2010). This process is initiated by specific Ca^2+^‐activated proteases, the calpains, which release sarcomeres by attacking their structural anchors at the Z‐disk so that they can be quantitatively degraded by the proteasome (Bullard et al. 1990; Alderton and Steinhardt 2000; Tidball and Spencer 2002; Goll et al. 2008; Shenkman et al. 2015). One of these anchors is the costamere, a subsarcolemmal protein complex that couples the sarcomere with the extracellular matrix (Ervasti 2003). Among other costameric proteins, talin, vinculin, focal adhesion kinase (FAK), and its phosphorylation at the tyrosine 397 site (FAK‐pY397) are critical for costamere turnover and integrity. These proteins are also excellent calpain substrates. As an example, calpain can very rapidly cleave talin creating 190 kDa C‐terminal and 47 kDa N‐terminal fragments that are no longer functional (Goll et al. 2003). Calpain activity is triggered by increased intracellular Ca^2+^ concentration. Likewise counter‐regulation is observed by a broad variety of molecules, for example, its specific endogenous inhibitor calpastatin, nitric oxide (NO) or synthetic inhibitors like calpeptin, all of them having shown to save muscle protein compared with an untreated control (Alderton and Steinhardt 2000; Koh and Tidball 2000; Tidball and Spencer 2002; Goll et al. 2003; Fareed et al. 2006; Shenkman et al. 2015). In the ovine model of RC tear, loss of contractile tissue is associated with an increase in the 190 kDa proteolytic fragments of talin, suggesting a potential role of calpain (Ruoss et al. 2017). However, no causal connection could so far be established among calpain activity, costamere cleavage, and loss of contractile tissue.

It was therefore the aim of this study to test the following hypotheses: First, release of a rotator cuff tendon leads to increased calpain activity in the ovine model. Second, inhibition of calpain activity with calpeptin and the NO promoter sildenafil decreases costamere cleavage and consequently muscle atrophy and myotendinous retraction.

## Methods

### Ethical approval

Experiments were approved by the Veterinary Office of the Canton of Zurich, Switzerland (No. ZH219/2014) and performed according to the Swiss law of animal welfare (TSchG455). Animal care and surgeries were performed by veterinarians at the Animal Hospital of the University of Zurich and animals’ pain and suffering were minimized by appropriate anesthesia and analgesia described previously (Gerber et al. 2004). Food and water were available ad libitum.

### Experimental design

In 18 female Swiss Alpine sheep (age: 26.7 ± 1.4 months [mean ± SD]), distal tendon tear of the *m. infraspinatus* was simulated by osteotomy of the greater tuberosity of the right shoulder as described previously (Gerber et al. 2015). Six sheep received the calpain inhibitor calpeptin (group CALP, body mass: 63.5 ± 5.5 kg). Another six sheep were administered daily doses of sildenafil additionally to calpeptin (group CALPSILD, body mass: 62.1 ± 1.9 kg) and the remaining six sheep served as CONTROL group by receiving the calpeptin vehicle (body mass: 63.3 ± 3.4 kg). Prior to tendon release (PRE) and after 2, 4, and 6 weeks, the right *m. infraspinatus* was biopsied to determine tissue composition, fiber type distribution, protein expression, and calpain activity. Furthermore, computed tomography (CT) scans were performed to assess muscle volume, length, and CSA at 30 min, 2, 4, and 6 weeks. The left shoulder was not manipulated until a single biopsy was taken before euthanasia at 6 weeks and served as a group‐internal contralateral control (CC). One sheep of the CALP group was euthanized at 2 weeks and excluded from analysis due to a fracture of the greater tuberosity. No further complications occurred and all other sheep were euthanized at 6 weeks.

### Administration of calpain inhibitor and sildenafil

Calpeptin (#ab120804, Abcam, Cambridge, UK) was dissolved in 75% dimethyl sulfoxide (DMSO) to a final concentration of 12.5 mg mL^−1^ and administered locally by a 2 mL Alzet^®^ osmotic pump (2ML4, Durect Corp., Cupertino CA) modified with a polyethylene catheter (PE‐60, Durect Corp.) attached to the PEEK™ flow moderator (Victrex PLC, Thornton Cleveleys, UK). Constant administration (pump flow rate: 2.5 *μ*L/h, calpeptin daily dose: 0.75 mg, DMSO daily dose: 45 *μ*L) was verified by recollecting the leftover volume after the pump was removed from the sheep.

One daily dose of 50 mg sildenafil (Pfizer^®^, New York) was administered in tablet form in the mornings. Entry through the gastrointestinal tract was confirmed by detecting increased intraocular pressure (Gerometta et al. 2010) using a TonoVet^®^ tonometer (Icare, Helsinki, Finland) in patient mode “p” according to the user manual. Values of oculus dexter (OD) and oculus sinister (OS) were pooled.

### Tendon release and implantation of the osmotic pump

The distal tendon of the right *m. infraspinatus* was released by chipping the bone piece of the greater tuberosity where the tendon inserts using an oscillating saw. To avoid spontaneous attachment, the tendon and bone chip were wrapped in a silicone tube as described previously (Gerber et al. 2015).

The Alzet^®^ pump was implanted at the caudal aspect of the scapular spine, cranially of the right *m. infraspinatus* and approximately 5 cm dorsal to the acromion. In detail, a 0.5 cm incision was made right at the crest of the scapular spine using a scalpel blade and a pocket was created with a periosteal elevator, so that the pump could be inserted and moved dorsally. The exit point of the catheter was distal, 6 cm dorsal from the acromion, and the pump was turned so that the natural curvature of the catheter pointed towards the muscle. A small incision was made into the muscle at approximately 3 cm distal to the scapular spine using a small curved mosquito clamp. A tiny tunnel was pierced through which the catheter was pulled into the muscle belly without being kinked. The catheter outlet was left freely in the distal third of the lateral aspect of the muscle. The pump was implanted in all sheep, filled with either calpeptin or vehicle only. To cover a time course of 6 weeks, the pump was replaced at 2 weeks as its volume allowed drug delivery for up to 4 weeks only. All surgeries were performed blinded.

### Sampling of muscle tissue

Muscle biopsies were collected intraoperatively with the sheep under general anesthesia. Approximately 50 mg of the distal, lateral third of *m. infraspinatus* was extracted using a 5 mm Bergstroem needle (Dixons Surgical Instruments LTD, Wickford), immediately frozen in liquid nitrogen cooled isopentane and stored at −80°C until analysis.

### Muscle volume, length, and cross‐sectional area

CT scans (Somatom ART; Siemens Medical Solutions, Erlangen, Germany) of both shoulders were performed with the sheep in supine position, under general anesthesia. The baseline measurement was conducted directly after surgery (i.e., 30 min after tendon release) and all follow‐up scans were performed before surgery. Axial images (slice thickness: 1 mm, tube voltage: 120 kV, tube current: 200 mA/slice) were acquired and reconstructed in the coronal plane. All measurements were performed blinded. Infraspinatus muscle volume and CSA were assessed on both sides using the free‐hand ROI (region of interest) tool in the Merlin PACS software (v5.121375, Phönix, Freiburg i. Br., Germany) as follows: Muscle volume was calculated from areas assessed on axial images at every 4 mm by drawing a ROI surrounding the outer muscle contour. CSA was measured on a standardized coronal slice defined as lying 1 cm anterior to the anterior end of the pump (where the catheter outlet was expected). Muscle length was measured at its longest diameter on axial slices from posterior to anterior including the distal tendon stump, but excluding the bone chip on the operated side, or until bone insertion on the contralateral side, respectively.

### Tissue distribution

Two consecutive 12 *μ*m thin sections were stained for either contractile and connective tissue using the Goldner trichrome technique (Goldner 1938) or for fat with oil red O as described (Ruoss et al. 2017). Total area (=100%) and fat area percentage were measured on the oil red O staining and contractile tissue area percentage on the Goldner‐stained section using Image J (v1.48v, National Institutes of Health) according to a standard procedure (Ruoss et al. 2017). The area percentage of non‐fatty connective tissue was calculated as “total area ‐ (fatty tissue area + contractile tissue area)”. The fiber type distribution was assessed by immunofluorescence staining of the slow and fast types myosin heavy chain (MyHC) isoforms 1 and 2, respectively, (protocol described previously (Ruoss et al. 2017)), using a mouse *α*‐MyHC‐1 (#MAB1628, Millipore Corp., Temecula, 1:100) and a rabbit *α*‐MyHC‐2 primary antibody (#ab91506, Abcam1:100), whereas fibers stained for both isoforms were specified as hybrid fibers. As secondary antibodies, the *α*‐mouse Alexa Fluor^®^ 488 #A11017 and anti‐rabbit Alexa Fluor^®^ 555 #A21428 (Thermo Fisher Scientific, Waltham) were used 1:200. All measurements were performed blinded.

We have recently shown in this model that the sole consideration of a % change in muscle composition may be misleading if the absolute volume to which it is related, undergoes alterations, too (Ruoss et al. 2017). Therefore, we also report approximate volumes of the assessed tissue and fiber types. They were calculated by extrapolation of the area percentages from histological results to the respective muscle volumes assessed on the CT scans.

### Calpain activity

Approximately 5 mg of tissue was homogenized in 115 *μ*L KH_2_PO_4_. (0.1 mol L^−1^, pH = 7.2), of which 25 *μ*g of total protein in KH_2_PO_4_ was loaded on a 96‐well plate (#655900, Greiner Bio‐One, Kremsmünster, Austria) and filled up to a total volume of 48 *μ*L with reaction buffer (60 mmol L^−1^ imidazole, pH = 7.3; 2% DMSO/MetOH; 2.5 mmol L^−1^ *β*‐mercaptoethanol; ddH_2_O) containing 250 *μ*mol L^−1^ Suc‐Leu‐Tyr‐Aminomethylcoumarin (#I1355, Bachem, Bubendorf, Switzerland) as substrate. A standard curve of free aminomethylcoumarin (AMC; #BML‐KI144, Enzo Life Sciences, Farmingdale NY) in reaction buffer was loaded on every plate. The fluorescence excitation (340/30 nm filter) was performed on a Synergy HT plate reader using Gen5 software (v2.09, BioTek Instruments, Winooski VT) and emission (460/40 nm filter) was recorded every 10 min. Enzymatic activity of calpain is expressed as slope of increasing free AMC concentration over time, that was determined from all reading points being located in the linear phase of the parabolic curve before plateauing, which was from 0 to 50 min for all 85 samples.

### Protein levels

Western blotting was performed as described previously (Ruoss et al. 2017). Target proteins were detected in 10 *μ*g of total protein homogenate using the Biorad Trans‐Blot Turbo System (Biorad, Cressier, Switzerland), only FAK‐pY397 was detected in 10 *μ*L of precipitate that was obtained from 250 *μ*g of total protein as described (Ruoss et al. 2017) using 200 *μ*g protein A‐sepharose (#P9424, Sigma‐Aldrich, St. Louis) and a combination of anti‐FAK‐pY397 antibodies (1 *μ*L each, #44‐624G, Thermo Fisher Scientific, Waltham; #sc‐11765‐R, Santa Cruz Biotechnology, Texas). The following primary antibodies were used: rabbit *α*‐FAK serum (1:1000) (Fluck et al. 1999), monoclonal mouse *α*‐talin #ab95034 (Abcam; 1:100), mouse *α*‐vinculin serum (gift of Dr. M. A. Glukhova (Glukhova et al. 1990), Paris, France; 1:100), rabbit *α*‐calpain1 #ab28258 (Abcam; 1:2000), rabbit *α*‐calpain2 #ab39165 (Abcam; 1:2000), and a monoclonal mouse *α*‐PPAR‐gamma #LS‐C178333 (Lifespan Biosciences Inc., LabForce AG, Nunnigen, Switzerland; 1: 1000). The goat *α*‐mouse #A9917 (Sigma‐Aldrich, St. Louis; 1:20000) and goat *α*‐rabbit #55676 (MP Biomedicals, Ohio; 1:20000) were used as secondary antibodies. Blotting quality and equal loading were verified using Ponceau S staining and target bands were quantified using the rectangular mode in the Quantity One software (Biorad, Cressier, Switzerland) according to the user manual. Inter‐membrane variability was controlled by dividing the signals by an internal standard that was loaded on every gel. Afterward, the relative changes of the signal intensities were related to the PRE‐values.

### Statistics

All data are presented as mean ± SD. SPSS Statistics v22.0 (IBM, Armonk) was used to run statistical analysis. Differences between time points and intervention groups were detected using a split‐plot ANOVA with “time point” (PRE, 2, 4, and 6 weeks) as repeated within‐subjects factor and “group” (CONTROL, CALP, CALPSILD) as between‐subjects factor. Comparison with the contralateral control was drawn using a split‐plot ANOVA with the repeated within‐subjects factor “shoulder” (operated, contralateral) and the between‐subjects factor “treatment.” Normal distribution was verified using a Shapiro‐Wilk test. If not normally distributed data were detected at a single time point within the time course, the split‐plot ANOVA was still performed as it has been shown to be robust against minor violations of normality as long as sample size does not change (Field 2009). The PPAR‐gamma 1 and 2 PRE‐values of one sheep in the CALP group were identified as outliers, being located more than 2.2x of the interquartile range above the upper quartile (Hoaglin and Iglewicz 1987). These two PRE‐values were excluded from PPAR‐gamma analysis. Statistical significance was defined as *P *<* *0.05.

## Results

### Calpeptin with and without sildenafil administration mitigates retraction and delays atrophy of the released rotator cuff muscle

**Figure 1:**
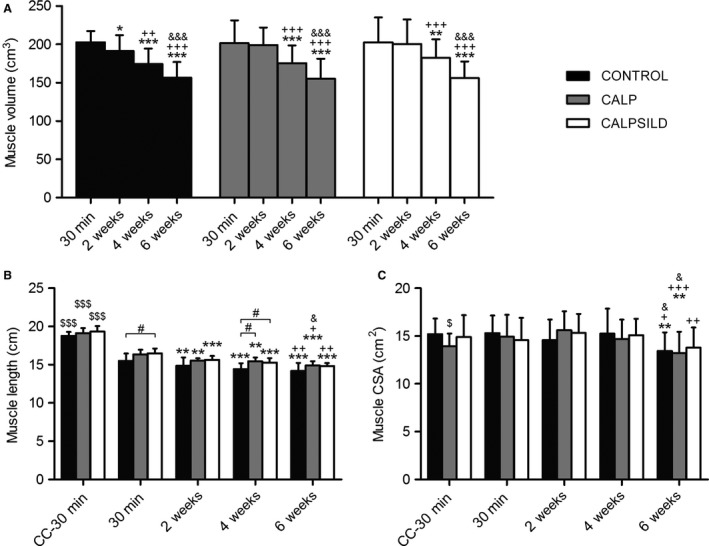
Calpain inhibition delays atrophy of rotator cuff muscle and mitigates shortening of the muscle‐tendon complex. Muscle morphometry of the operated *m. infraspinatus* and the contralateral control (CC) assessed at 30 min, 2, 4, and 6 weeks after surgical tendon release. (A) Loss of muscle volume started between 2 and 4 weeks in the calpeptin‐treated sheep (CALP,* n *=* *5; CALPSILD,* n *=* *6), while it was already detected at 2 weeks in the CONTROL group (*n *=* *6). (B) Calpeptin treatment acutely mitigated shortening of the muscle. (C) Muscle cross‐sectional area (CSA) did not decrease before 6 weeks. Time effects: * *P *<* *0.05, ** *P *<* *0.01, *** *P *<* *0.001 versus 30 min; ^+^ *P *<* *0.05, ^++^ *P *<* *0.01, ^+++^ *P *<* *0.001 versus 2 weeks; ^&^ *P *<* *0.05, ^&&&^ *P *<* *0.001 versus 4 weeks. Group effects: ^#^ *P *<* *0.05 versus CONTROL;* P* < 0.05^, $$^ *P *<* *0.001 versus operated shoulder at 30 min. Bars are means ± SD.

All intervention groups started with a similar *m. infraspinatus* volume (CONTROL: 202.6 ± 14.8 cm^3^; CALP: 201.5 ± 29.8 cm^3^; CALPSILD: 202.6 ± 32.5 cm^3^, Figure 1A. Volumes of the contralateral *m. infraspinatus* are displayed in Fig. S1A). Analysis of CT scans from 30 min, 2, 4, and 6 weeks after tendon release revealed that calpeptin administration prevented muscle atrophy for 2 weeks while muscle volume was lost in the CONTROL group (−11.6 ± 9.1 cm^3^, *P *=* *0.038; Fig. 1A). The protective effect of calpain inhibition was gone at 4 weeks and 6 weeks as muscle atrophy was detected in all groups compared to 30 min (4 weeks: CONTROL: −28.4 ± 14.6 cm^3^, *P *<* *0.001; CALP: −26.2 ± 15.0 cm^3^, *P *< 0.001; CALPSILD: −20.1 ± 10.7 cm^3^, *P *=* *0.003; 6 weeks: CONTROL: −45.8 ± 15.8 cm^3^, *P *<* *0.001; CALP: −46.3 ± 10.8 cm^3^, *P *<* *0.001; CALPSILD: −46.7 ± 12.0 cm^3^, *P *<* *0.001; Fig. 1A). In parallel, immediate retraction of the *m. infraspinatus* was diagnosed in all groups compared with the contralateral side and further progressed over the 6 weeks time course (Fig. 1B). Compared with the CONTROL group, the muscle was significantly longer at 30 min after tendon release in the CALPSILD (+1.0 cm, *P *=* *0.042), but not in the CALP group (+0.9 cm, *P *=* *0.083); while at 4 weeks, it was significantly longer in both CALPSILD (+0.8 cm, *P *=* *0.040) and CALP groups (+1.0 cm, *P *=* *0.020; Fig. 1B). Muscle CSA assessed close to the drug delivery site, was significantly decreased in CONTROL and CALP (−1.9 ± 1.4 cm^2^, *P *=* *0.001 and −1.7 ± 1.1 cm^2^, *P *=* *0.005, respectively), but not in the CALPSILD group (−0.8 ± 0.9 cm^2^, *P *=* *0.123) at 6 weeks compared with 30 min after tendon release (Fig. 1C).

### Calpeptin delays early slow‐to‐fast phenotype shift

**Figure 2:**
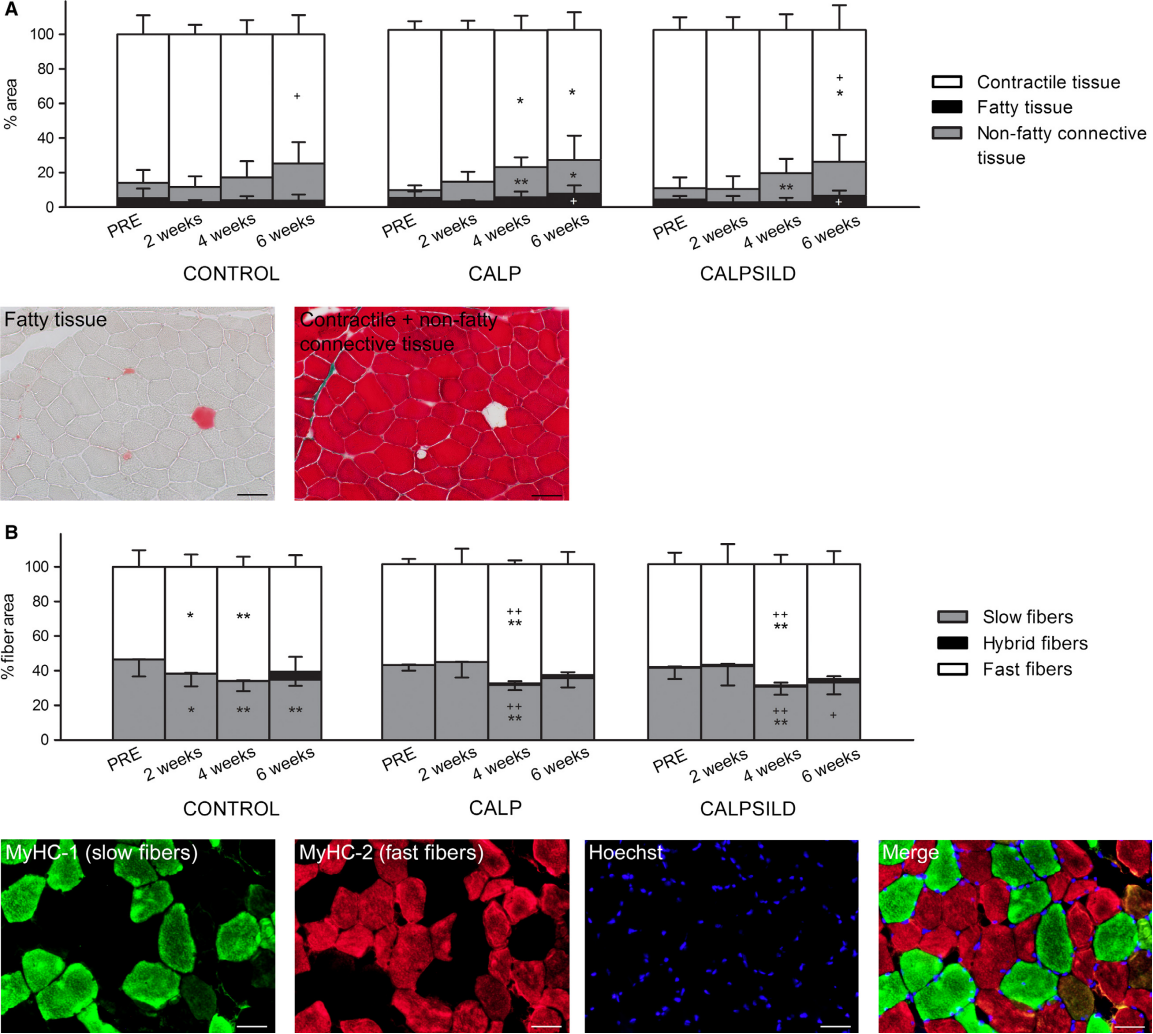
Tendon release leads to changes in relative contractile versus non‐contractile tissue distribution and to a slow‐to‐fast fiber phenotype shift, of which the latter is delayed by calpain inhibition. The distribution of different tissue types assessed prior to, and 2, 4, and 6 weeks after tendon release. (A) Loss of contractile tissue and fibrosis were not detected before 2 weeks after tendon release. (B) Fiber type distribution assessed on myosin heavy chain (MyHC) isoforms 1 (green, slow) and 2 (red, fast) stained sections. Hybrid fibers express both isoforms and appear orange on the merge image. The slow‐to‐fast phenotype shift was detected from 2 weeks on in the CONTROL group (*n *=* *6), but not until 4 weeks with calpain inhibition (CALP,* n *=* *5; CALPSILD,* n *=* *6). Time effects: * *P *<* *0.05, ** *P *<* *0.01 versus PRE; ^+^ *P *<* *0.05, ^++^ *P *<* *0.01 versus 2 weeks. Columns are means + SD. Black scale bars denote 100 *μ*m; white scale bars denote 50 *μ*m.

**Figure 3:**
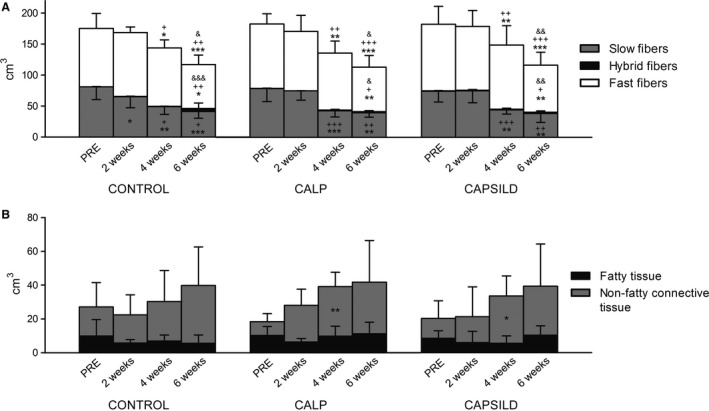
Early atrophy after 2 weeks is explained by loss of slow myofibers. Volumes of the different tissue types assessed prior to, and 2, 4, and 6 weeks after tendon release (A) Fiber volumes revealed that the loss of slow fibers was delayed by calpain inhibition while no quantitative degradation of fast fibers was detected before 6 weeks. (B) Volumes of fatty and non‐fatty connective tissue were altered similarly in the CONTROL (*n *=* *6) and the calpain inhibition groups (CALP,* n *=* *5, CALPSILD,* n *=* *6). Time effects: * *P *<* *0.05, ** *P *<* *0.01, *** *P *<* *0.001 versus PRE; ^+^ *P *<* *0.05, ^++^ *P *<* *0.01, ^+++^ *P *<* *0.001 versus 2 weeks; ^&^ *P *<* *0.05, ^&&^ *P *<* *0.01, ^&&&^ *P *<* *0.001 versus 4 weeks. Indications above columns refer to significant differences in total contractile tissue volume; indications inside columns refer to significant differences in the specific fiber type volume. Columns are means + SD.

Total contractile tissue area was not affected at 2 weeks (Fig. 2A), but the area occupied by slow fibers significantly decreased at 2 weeks compared with PRE in the CONTROL group (−8.3 ± 10.7%, *P *=* *0.046). Slow fiber area percentage remained unchanged in the CALP (+1.8 ± 9.4%, *P *=* *0.675) and CALPSILD groups (+1.1 ± 7.6%, *P *=* *0.777; Fig. 2B). After 4 weeks, a relative decrease in slow fiber area percentage was detected in all groups (CONTROL: −12.5 ± 9.6%, *P *=* *0.002; CALP: −11.2 ± 4.8%, *P *=* *0.006; CALPSILD: −10.7 ± 7.6%, *P *=* *0.005; compared with the baseline) and being scaled to a total fiber area of 100%, it is logical that these effects were paralleled by a relative increase in the fast fiber area percentage (Fig. 2B). Then, we extrapolated this relative distribution to the contractile tissue volume (see Methods) and found the increase in the fast fiber portion not confirmed. On the contrary, fast fiber volume remained unchanged in all groups until 4 weeks and was significantly decreased at 6 weeks compared with baseline (CONTROL: −23.5 ± 28.8 cm^3^, *P *=* *0.023; CALP: −32.2 ± 11.7 cm^3^, *P *=* *0.007; CALPSILD: −31.5 ± 22.0 cm^3^, *P *=* *0.004; Fig. 3A). Slow fiber volume was decreased at 2 weeks in the CONTROL group (−8.3 ± 10.7 cm^3^, *P *=* *0.026), but not in the calpeptin‐treated sheep (CALP: +1.8 ± 9.4 cm^3^, *P *=* *0.605; CALPSILD: +1.1 ± 7.6 cm^3^, *P *=* *0.931) compared with PRE (Fig. 3A). Neither the area percentage nor the volume of slow/fast hybrid fibers were significantly increased (Fig. 2B and 3A).

### Tendon release leads to an early modulation of fatty and non‐fatty connective tissue

Fatty tissue area percentage tended to decrease over all groups at 2 weeks (−1.9 ± 3.9%, *P *=* *0.084, compared with PRE). Between 2 weeks and 6 weeks, it increased in the calpeptin‐treated sheep (CALP: +4.4 ± 4.8%, *P *=* *0.017; CALPSILD: +3.5 ± 2.1%, *P *=* *0.033; Fig. 2A). Over all groups, fatty tissue volume was significantly larger at 6 weeks than at 2 weeks (+3.0 ± 5.8 cm^3^, *P *=* *0.041; Fig. 3B).

The area percentage of nonfatty connective tissue increased from 4 weeks onwards in the calpeptin‐treated sheep (CALP: +12.8 ± 5.0%, *P *=* *0.002; CALPSILD: +9.7 ± 4.3%, *P *=* *0.006; Fig. 2A). The respective volume was not significantly altered after tendon release in the CONTROL group, but increased at 4 weeks compared with baseline in both CALP (+21.1 ± 7.7 cm^3^, *P *=* *0.004) and CALPSILD groups (+16.3 ± 6.9 cm^3^, *P *=* *0.010; Fig. 3B).

### Tendon release increases calpain activity, which is blunted under calpeptin administration

**Figure 4:**
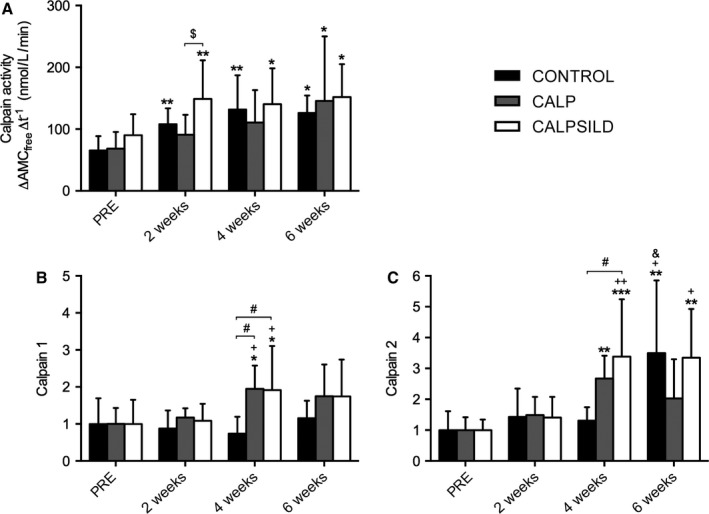
Blunting of calpain activity after tendon release is counter‐regulated by increasing calpain protein concentrations. Calpain activity is expressed as the change in concentration of the cleaved aminomethylcoumarin (AMC_free_) per time. Protein levels were assessed in biopsy tissue that was harvested prior to, and 2, 4, and 6 weeks after tendon release. (A) Tendon release increased calpain activity (CONTROL,* n *=* *6), which was blunted by calpeptin treatment alone (CALP,* n *=* *5), but not in combination with sildenafil (CALPSILD,* n *=* *6). (B) Calpain 1 and 2 concentrations were upregulated after 4 weeks in the calpeptin‐treated groups only. Time effects: * *P *<* *0.05, ** *P *<* *0.01, *** *P *<* *0.001 versus PRE; ^+^ *P *<* *0.05 versus 2 weeks; ^&^ *P *<* *0.05 versus 4 weeks. Group effects: ^#^ *P *<* *0.05 versus CONTROL; ^$^ *P *<* *0.05 versus CALP. Bars are means ± SD.

Calpain activity (Fig. 4A) increased at all assessed time points compared with PRE in CONTROL (2 weeks: +42.5 ± 19.0 nmol L^−1^ min^−1^, *P *=* *0.010; 4 weeks: +66.1 ± 34.1 nmol L^−1^ min^−1^, *P *=* *0.006; 6 weeks: +60.6 ± 20.7 nmol L^−1^ min^−1^, *P *=* *0.032) and CALPSILD (2 weeks: +58.7 ± 51.5 nmol L^−1^ min^−1^, *P *= 0.001; 4 weeks: +50.2 ± 70.3 nmol L^−1^ min^−1^, *P *= 0.026; 6 weeks: +61.7 ± 74.4 nmol L^−1^ min^−1^, *P *=* *0.030). In the CALP group, a significant increase in calpain activity was only detected at 6 weeks compared with baseline (2 weeks: +22.6 ± 21.9 nmol L^−1^ min^−1^, *P *=* *0.169; 4 weeks: +42.2 ± 30.8 nmol L^−1^ min^−1^, *P *=* *0.077; 6 weeks: +77.3 ± 78.6 nmol L^−1^ min^−1^, *P *=* *0.015; Fig. 4A). Calpain activity in the contralateral side remained unaffected (Fig. S1B).

### Inhibition of calpain activity is counter‐regulated by increased calpain protein content

**Figure 5:**
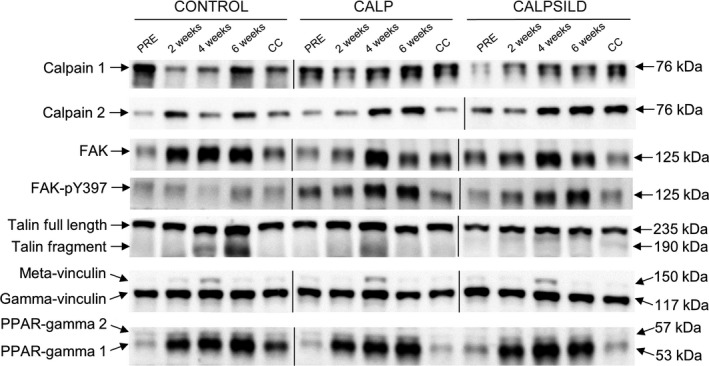
Representative western blot images. Calpain 1, calpain 2, focal adhesion kinase (FAK), phosphorylated FAK (FAK‐pY397), talin, gamma‐ and meta‐vinculin, PPAR‐gamma 1 + 2 assessed in muscle tissue biopsied prior to, and 2, 4, and 6 weeks after surgical tendon release. The contralateral control shoulder (CC) was biopsied at 6 weeks. This figure is assembled from two representative membranes per target protein.

Calpain 1 protein levels remained unaffected in the CONTROL group and were significantly increased in CALP (+94.7 ± 89.6%, *P *=* *0.024) and CALPSILD (+91.7 ± 89.1%, *P *=* *0.018) after 4 weeks. The increase at 6 weeks was statistically not significant (CALP: +74.9 ± 108.4%, *P *=* *0.100; CALPSILD: +74.6 ± 106.5%, *P *=* *0.075; Fig. 4B). Calpain 2 levels increased likewise in the calpeptin‐treated groups at 4 weeks (CALP: +167.3 ± 89.6%, *P *=* *0.008; CALPSILD: +238.2 ± 162.7%, *P *<* *0.001) with the CONTROL group remaining at baseline level (Fig. 4C). At 6 weeks, calpain 2 levels increased in the CONTROL (+249.8 ± 234.2%, *P *= 0.006) and CALPSILD groups (+234.8 ± 170.4%, *P *= 0.009), but not in the CALP group (+102.6 ± 139.2%, *P *=* *0.244; Fig. 4C). Representative western blot images are shown in Fig. 5.

### Calpeptin prevents from the unloading‐induced decrease in relative FAK phosphorylation and cleavage of talin

**Figure 6:**
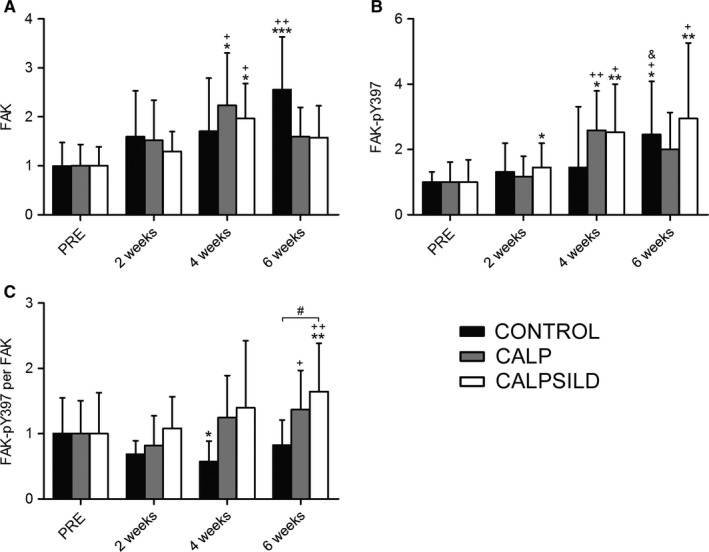
Calpain inhibition increases focal adhesion kinase (FAK) and FAK‐pY397 concentrations after tendon release. Relative protein levels were assessed prior to, and 2, 4, and 6 weeks after tendon release in calpeptin‐treated (CALP,* n *=* *5), calpeptin and sildenafil‐treated (CALPSILD,* n *=* *6), and untreated sheep (CONTROL,* n *=* *6). (A) FAK levels increased after tendon release. (B) FAK‐pY397 levels increased with calpain inhibition, but remained unchanged in the CONTROL group for the first 4 weeks. (C) Specific FAK‐Y397 levels (FAK‐pY397 per FAK) decreased after tendon release, which was prevented by calpain inhibition. Time effects: * *P *<* *0.05, ** *P *<* *0.01, *** *P *<* *0.001 versus PRE; ^+^ *P *<* *0.05 versus 2 weeks; ^&^ *P *<* *0.05 versus 4 weeks. Group effects: ^#^ *P *<* *0.05 versus CONTROL. Bars are means ± SD.

FAK (Fig. 6A) and FAK‐pY397 (Fig. 6B) were variably affected, leading to decreased specific FAK‐pY397 levels (FAK‐pY397 per FAK) after tendon release in the CONTROL group (2 weeks: −31.4 ± 53.0%, *P *=* *0.061; 4 weeks: −42.6 ± 35.7%, *P *=* *0.049; 6 weeks: −17.7 ± 29.8%, *P *= 0.302), while it remained unaffected until 4 weeks and was increased at 6 weeks in both CALP and CALPSILD (Fig. 6C).

**Figure 7:**
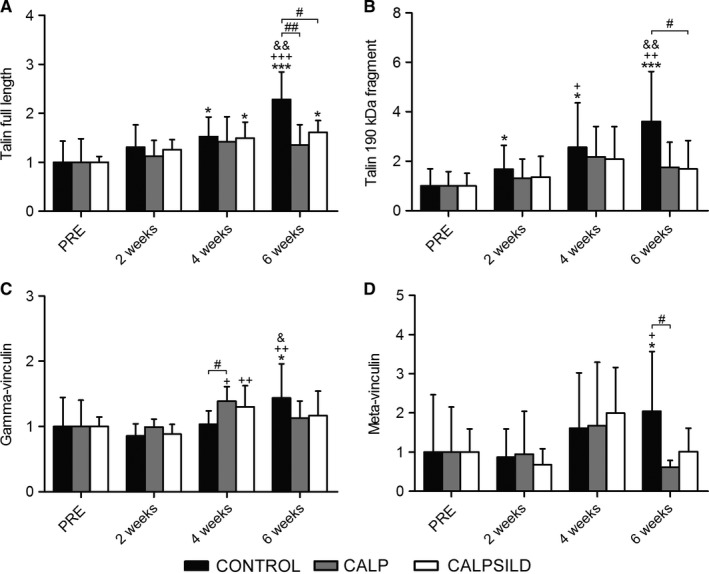
Calpain inhibition prevents cleavage of talin into a 190 kDa proteolytic fragment after tendon release. Relative protein levels assessed prior to, and 2, 4, and 6 weeks after tendon release in calpeptin‐treated (CALP,* n *=* *5), calpeptin and sildenafil‐treated (CALPSILD,* n *=* *6), and untreated sheep (CONTROL,* n *=* *6). (A) The increase in full length talin was more pronounced in the CONTROL than in the treatment groups despite a higher cleavage rate, which is indicative for an increased costamere turnover. (B) The cleaved C‐terminal proteolytic 190 kDa fragment of talin increased after tendon release and cleavage was prevented by calpain inhibition. (C) Gamma‐vinculin, a main structural protein of the costamere, altered similarly to FAK and may, together with full length talin, represent alterations in costamere abundance. (D) Meta‐vinculin was very variably expressed both at baseline and after tendon release. Time effects: * *P *<* *0.05, *** *P *<* *0.001 versus PRE; ^+^ *P *<* *0.05, ^++^ *P *<* *0.01, ^+++^ *P *<* *0.001 versus 2 weeks; ^&^ *P *<* *0.05, ^&&^ *P *<* *0.01 versus 4 weeks. Group effects: ^#^ *P *<* *0.05, ^##^ *P *<* *0.01 versus CONTROL. Bars are means ± SD.

Full length talin protein increased in the CONTROL (4 weeks: +52.5 ± 44.1%, *P *=* *0.031; 6 weeks: +128.2 ± 61.0%, *P *<* *0.001; compared with PRE) and CALPSILD groups (4 weeks: +49.4 ± 38.9%, *P *=* *0.041; 6 weeks: +60.5 ± 29.9%, *P *=* *0.015; compared with PRE; Fig. 7A) and the proteolytic 190 kDa fragment of talin significantly increased in the CONTROL group after tendon release (2 weeks: +68.4 ± 70.3%, *P *=* *0.014; 4 weeks: +156.4 ± 177.9%, *P *=* *0.011; 6 weeks: +261.1 ± 217.5%, *P *<* *0.001; compared with PRE) which was prevented by calpain inhibition (Fig. 7B). Gamma‐vinculin levels increased in the calpeptin‐treated sheep at 4 weeks (Fig. 7C). Meta‐vinculin levels varied between sheep and only the increase in the CONTROL group at 6 weeks was statistically significant (+104.3 ± 131.2%, *P *=* *0.039, compared with PRE; Fig. 7D).

### PPAR‐gamma isoforms 1 and 2 expression is unaffected by calpain inhibition

**Figure 8:**
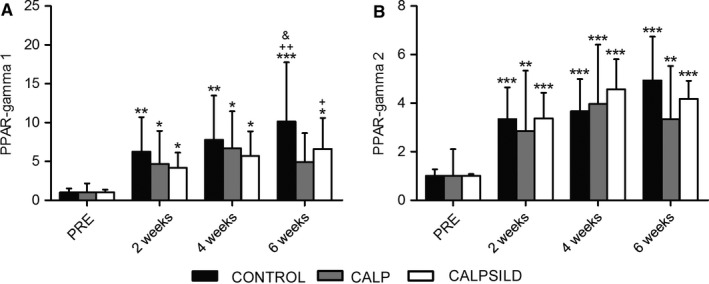
PPAR‐gamma 1 and 2 protein levels increase after tendon release. Relative protein levels were assessed prior to, and 2, 4, and 6 weeks after tendon release in calpeptin‐treated (CALP,* n *=* *5), calpeptin and sildenafil‐treated (CALPSILD,* n *=* *6), and untreated sheep (CONTROL,* n *=* *6). Both (A) PPAR‐gamma 1 and (B) PPAR‐gamma 2 isoforms dramatically increased. Time effects: * *P *<* *0.05, ** *P *<* *0.01, *** *P *<* *0.001 versus PRE; ^+^ *P *<* *0.05, ^++^ *P *<* *0.01 versus 2 weeks; ^&^ *P *<* *0.05 versus 4 weeks. Bars are means ± SD.

PPAR‐gamma 1 and 2 isoforms significantly increased in all groups at all time points by at least +319% and +186%, respectively, (Fig. 8).

### Sildenafil increases intraocular pressure in sheep

At 2 h after sildenafil administration, intraocular pressure (pooled values of *oculus dexter* and *oculus sinister*) was elevated (+2.1 ± 2.2 mmHg, *P *=* *0.004) and stayed above resting level at 4 h (+2.2 ± 1.5 mmHg, *P *=* *0.005; Fig. S2). A significant group difference was detected at 2 h compared with the untreated sheep (+2.1 mmHg, *P *=* *0.022). No side effects of sildenafil were observed.

## Discussion

Recovery from a chronic RC tear still poses a clinical challenge once muscle atrophy, myotendinous retraction, and fat accumulation have established. This study tested a novel approach to prevent atrophy and retraction after RC tendon release in sheep with the hope to indicate a new potential research orientation for the treatment of human RC tears. We found that inhibition of the calcium‐activated protease calpain counteracted costamere cleavage and delayed muscle atrophy, retraction, and slow‐to‐fast phenotype shift until counter‐regulation took place by increased calpain 1 + 2 protein levels. This suggests that the protease calpain may play an important role in the early degradation of contractile tissue after RC tendon release in sheep.

Atrophy, retraction, and intramuscular fat content are the best predictors for successful surgical repair and subsequent recoverability of chronic RC tear (Bartolozzi et al. 1994; Goutallier et al. 1994; Thomazeau et al. 1997; Gladstone et al. 2007; Oh et al. 2009). This study demonstrates that two of these three predictors can be tackled by calpain inhibition. Atrophy was prevented for 2 weeks by treatment with calpeptin alone and in combination with sildenafil (Fig. 1A). Microscopic measurements of tissue composition revealed that the main reason for atrophy in the untreated sheep was the loss of slow fiber volume and that this deterioration was prevented with calpain inhibition (Fig. 3B). The fiber degradation machinery, that is the proteasome system, requires calpain to disrupt structural anchors responsible for fiber integrity, so that the disorganized sarcomeric filaments can be ubiquitinated and digested (Smith and Dodd 2007; Goll et al. 2008). This is supported by our assessments of talin, a main structural anchor protein, which is cleaved into the 190 kDa C‐terminal proteolytic fragment by calpain (Hayashi et al. 1999; Goll et al. 2003; Smith and Dodd 2007) and was shown previously in a rat model of hindlimb unloading (Shenkman et al. 2015). The levels of this talin fragment significantly increased with tendon release alone, but not with concomitant inhibition of calpain (Fig. 7B). Interestingly, the preventive effect of calpain inhibition was abolished from 4 weeks onward, when atrophy, characterized by a reduction in the slow fiber portion, did not differ anymore between intervention groups. At this time point, our data suggests that the muscle overcame the inhibition by increasing calpain 1 and 2 enzyme levels (Fig. 4B + C). As already small delays of repair may have a detrimental impact on functional recovery in humans (Bassett and Cofield 1983; Petersen and Murphy 2011), the delay of atrophy for 2 weeks may already be clinically relevant, but this needs to be evaluated along with how this time window may further be extended.

From 2 weeks onward, retraction (i.e., shortening of the muscle‐tendon complex) is simply the manifestation of longitudinal atrophy and predominantly caused by loss of sarcomeres in series (Jamali et al. 2000; Ward et al. 2010), and thus may be explained by the same mechanism that led to decreased total muscle volume. The surprising finding is that calpeptin seemed to partially mitigate shortening at a time when quantitative loss of sarcomeres was not yet expected, that is, at 30 min after tendon release (Fig. 1B). Shortening at this time point has been shown to be the result of acutely decreased sarcomere length as opposed to sarcomere number (Jamali et al. 2000). The mechanism by which calpeptin may affect this acute retraction is largely unknown. However, its capacity to lower intracellular Ca^2+^ ‐concentration under circumstances where the sarcomere is tetanically contracted (Chin and Allen 1996) may play a role.

Two weeks of muscle unloading led to a slow‐to‐fast shift of the muscle phenotype, which was prevented by calpeptin (Fig. 2B). It is well documented that unloading leads to a change in MyHC isoform expression towards fast muscle (Andersen and Aagaard 2000; Trappe et al. 2004; Biering‐Sorensen et al. 2009) and it was reported before, that modulating calpain activity may mitigate slow‐to‐fast fiber conversion (Tidball and Spencer 2002). Though the exact mechanisms remain unclear, we speculate that prevention of fiber type switch and atrophy went hand in hand, as the thick type 1 myosin filament is tightly embedded within the thin actin filaments and connected to the Z‐disk via titin (*nota bene* an excellent calpain substrate (Goll et al. 2003)), and needed to be cut free first in order to be replaced by a myosin type 2 filament. Furthermore, it is conceivable that calpeptin‐induced differences in Ca^2+^ concentrations (Chin and Allen 1996) affected fiber type distribution via the CaN‐NFAT pathway (calcineurin ‐ nuclear factor of activated T cells) (Spangenburg and Booth 2003), or that calpeptin itself had an unknown direct effect on the expression of different MyHC isoforms. Like in atrophy, the preventive effect of calpeptin was gone after 4 weeks, maybe because calpain 1 + 2 enzyme concentrations doubled (Fig. 4B+C). Intriguingly, the calculation of approximate fiber volumes revealed that the fast fiber volume was not significantly reduced until at least 4 weeks (Fig. 3B). The fact that this was the case with and without calpeptin treatment, suggests that the degradation machinery preferred to attack slow over fast fibers during tendon tear‐induced muscle disuse, at least for the first 4 weeks. It is also possible that fast fiber digestion was compensated by a slow‐to‐fast conversion of fibers, although slow/fast hybrid fibers did not alter until 4 weeks and the seeming increase at 6 weeks was statistically not confirmed (Fig. 2B and 3B). Conversely, the loss of contractile tissue between 4 and 6 weeks was predominantly explained by decreased fast but not slow fiber volume. This observation was independent of drug treatment and calpain concentrations (compare Fig 3B with 4B + C) and may indicate that other processes which no longer reflect adaptations to unloading, have started to degenerate the muscle, because at 6 weeks, the typical unloading‐induced slow‐to‐fast conversion has stopped and the relative fast fiber portion is surprisingly not different from PRE‐values anymore (Fig. 2B).

Aside from atrophy, a massive increase in intramuscular fat content is observed in chronic RC tears. Also, the pathology and time course behind this adaptation is still hotly debated (Meyer and Ward 2016). Our recent study in sheep suggested that fatty tissue decreases first before quantitative accumulation occurs (Ruoss et al. 2017). Although the time course in the present study pointed to the same direction (Fig. 2A and 3A), this was statistically not confirmed, probably because the initial fat content was about 40% lower than in the previously investigated sheep (Ruoss et al. 2017) and therefore the potential for a relevant decrease was compromised. However, we found that the isoforms 1 and 2 of the adipocyte differentiation marker PPAR‐gamma were increased at 2–6 weeks after tendon release. PPAR‐gamma 1 represents a composite increase in adipocytes, preadipocytes, muscle‐derived stem cells (MDSCs) and/or other cell types (such as macrophages), and PPAR‐gamma 2 indicates an increase in mature adipocytes (Rosen and MacDougald 2006). Our PPAR‐gamma assessments are not compatible with the finding of little to no fat accumulation until 6 weeks (compare Fig. 8 with Fig. 2A + 3A). Either the alterations of fatty tissue fraction assessed on oil red O stained sections are below detection level, or PPAR‐gamma protein levels represent initiation of the adipocyte differentiation machinery and the adipocytes are yet to come. Both seem unlikely because Zhang and colleagues (Zhang et al. 2017) measured increased lipids using the oil red O technique already at 24 h after they induced fat cell differentiation in isolated bovine MDSCs. Thus, further study is needed to test the validity of PPAR‐gamma as a marker for fat cell differentiation in the ovine model of RC tears.

During myofibrillar turnover, calpains are known to make a few very selective cleavages and leave the job of quantitative degradation to the proteasome (Goll et al. 2008). Costamere components are amongst the target proteins of calpain (Goll et al. 2003) and due to their role as structural anchors for myofibers (Ervasti 2003), they may significantly influence the rate of myofibrillar degradation. We previously found that mitigating disruption of the costamere (i.e., mitigating cleavage of talin and relative dephosphorylation of the costamere‐stabilising FAK with anabolic steroids) may contribute to protection of contractile tissue after tendon release (Ruoss et al. 2017). The present study now specifically addressed preservation of the costamere complex through inhibition of calpain. Chronic local calpeptin administration prevented from relative dephosphorylation of FAK (Fig. 6C) and cleavage of talin into the calpain‐specific 190 kDa fragment (Fig. 7B). Interestingly, the ex‐vivo measurement of calpain activity was not mirroring calpain protein expression (Fig. 4), probably because regulation of calpain activity is a complex interplay between intracellular Ca^2+^ dynamics, calpain autolysis, calpastatin expression, and access to substrate (Goll et al. 2003). Furthermore, dynamics of other proteolytic systems that can break down myofibrils, such as the caspases (triggered by apoptotic signals) or the proteasome may affect costamere and contractile tissue turnover.

Although this study successfully showed the efficacy of calpain inhibition on atrophy in the ovine model of RC tears, there were several limitations. In order to allocate intervention effects of calpeptin (dissolved in 75% DMSO), the CONTROL group had to receive 75% DMSO. DMSO enhances membrane permeability by destabilizing the boundaries between the water and lipid bilayer, and thus may exert effects itself on skeletal muscle and its lipid content. However, compared to our recent ovine study of RC tear (Ruoss et al. 2017), the CONTROL group did show a similar grade of atrophy, intramuscular fat, and FAK and talin dynamics at 2 weeks after tendon release. Furthermore, the obtained biopsy specimen does not necessarily represent the whole muscle. Although the relative tissue distribution, calpain activity, and protein quantification precisely represent the analyzed piece of muscle, any extrapolations to the total volume should be understood as approximate values. Lastly, potential effects on skeletal muscle (and/or on the whole organism) by calpain inhibition, by calpeptin itself entering the circulation, and by sildenafil are manifold. However, we did not observe decreased calpain activity in the untreated contralateral shoulder (Fig. S1B). but many potential (side) effects may have been missed.

Concluding, this study enriches the understanding of early adaptations of RC muscle to simulated tendon tear in sheep. There is evidence for an early calpain‐mediated degradation of the costameric attachments and of the slow fibers. While during the first 2 weeks, atrophy does not differ from adaptions to milder forms of unloading (e.g., detraining), the typical slow‐to‐fast transformation with unloading has stopped at 6 weeks, and contractile tissue is lost to an extent that justifies the term “RC disease” although massive fat accumulation is not yet present. Inhibition of calpain prevented the early unloading adaptations, but not the subsequent initiation of RC disease. Further studies are needed to evaluate the clinical potential of calpain inhibition to mitigate atrophy and retraction, and to extend the time window before successful repair and recovery from a RC tear turn impossible.

## Conflict of Interests

The authors have declared that no conflict of interest exists.

## Data Accessibility

## Supporting information




**Figure S1.** Effects of tendon release and pharmacological treatment on the contralateral *m. infraspinatus*.

**Figure S2.** Gastrointestinal uptake of sildenafil was confirmed by detecting increased intraocular pressure.
