# Supplementary figures and images for: Inhibition of calpain delays early muscle atrophy after rotator cuff tendon release in sheep
Source: Physiol Rep. 2018 Nov 4;6(21):e13833. doi: 10.14814/phy2.13833 (PMC6215759; doi:10.14814/phy2.13833)

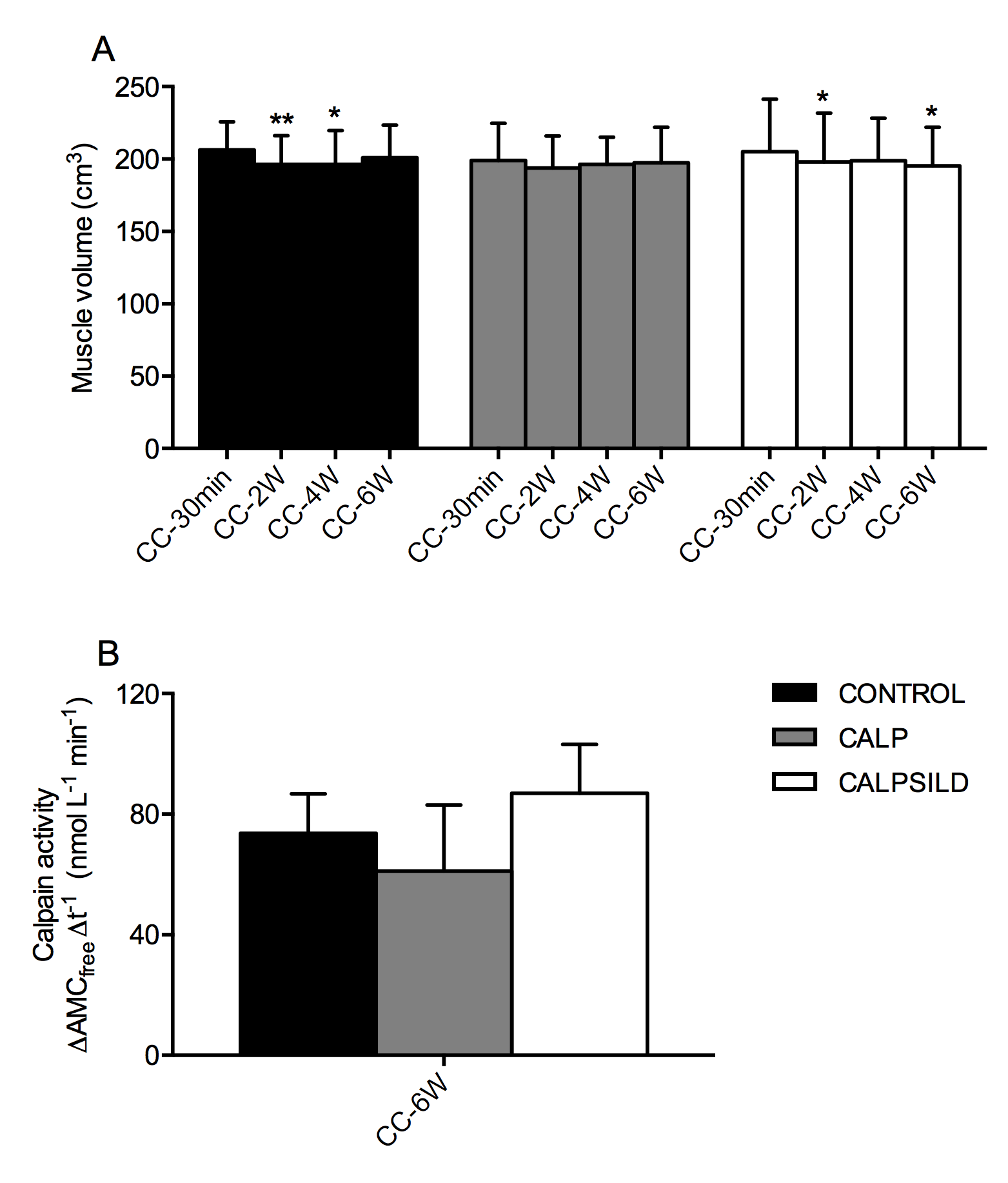

Supplement: Supplementary file 1 — Figure S1. Effects of tendon release and pharmacological treatment on the contralateral m. infraspinatus. [file PHY2-6-e13833-s001.tiff]

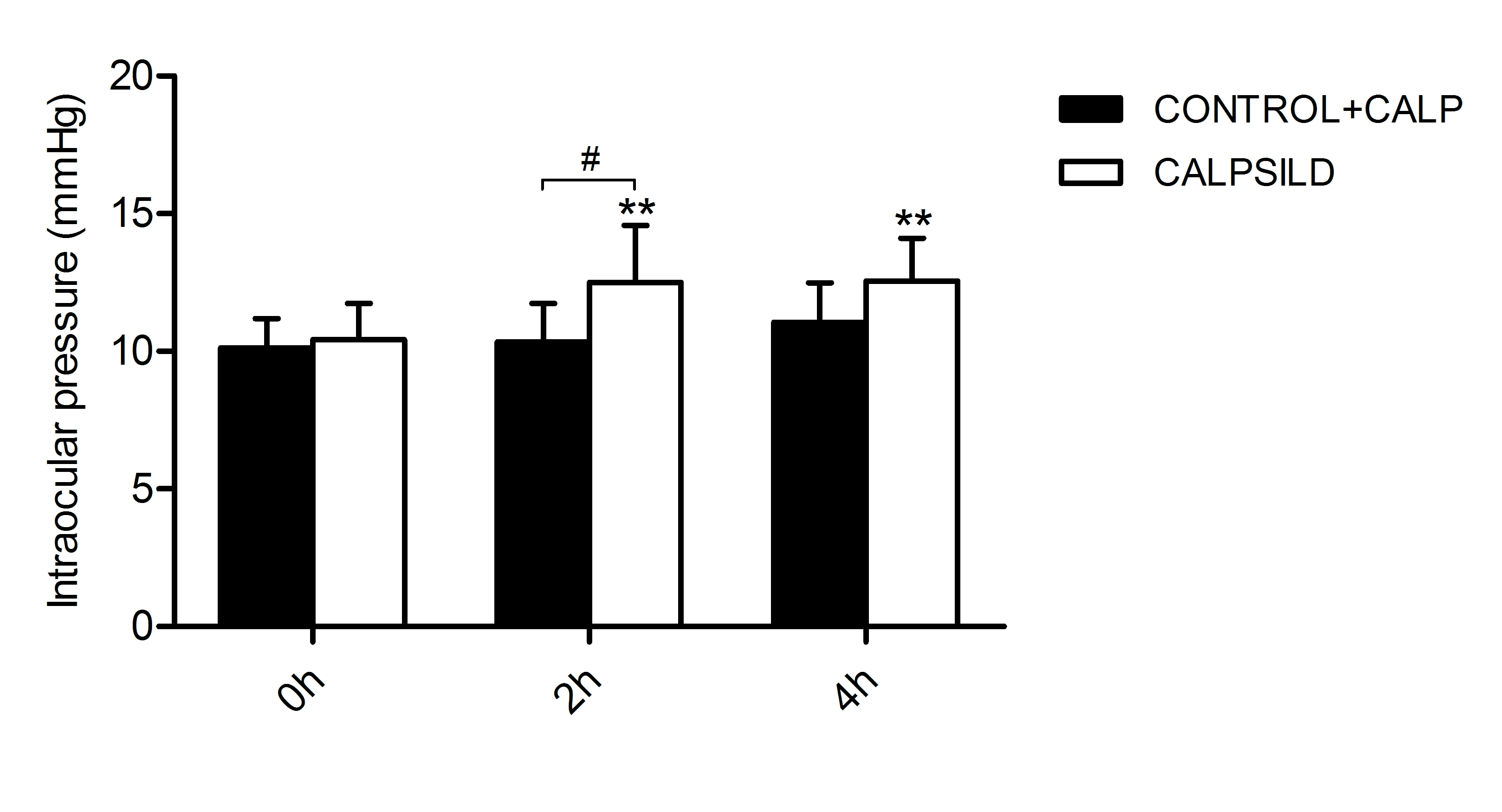

Supplement: Supplementary file 2 — Figure S2. Gastrointestinal uptake of sildenafil was confirmed by detecting increased intraocular pressure. [file PHY2-6-e13833-s002.tif]
