# Supplementary material for: Inhibition of calpain delays early muscle atrophy after rotator cuff tendon release in sheep
Source: Physiol Rep. 2018 Nov 4;6(21):e13833. doi: 10.14814/phy2.13833 (PMC6215759; doi:10.14814/phy2.13833)
Supplement: Supplementary file 3 [file PHY2-6-e13833-s003.docx]

**Fig. S1. Effects of tendon release and pharmacological treatment on the contralateral *m. infraspinatus*. (A)** Muscle volume of the left, contralateral control (CC) *m. infraspinatus* assessed at 30 min, 2, 4, and 6 weeks. **(B)** Calpain activity in the left, contralateral muscle was not affected by tendon release of the right muscle alone (CONTROL, n = 6) nor with calpeptin treatment (CALP, n = 5; CALPSILD, n = 6) after 6 weeks compared with the PRE-value of the manipulated side (compare with Fig. 4A). Time effects: * *p* < 0.05, ** *p* < 0.01, vs. CC-30min. Bars are means ± SD.

**Fig. S2. Gastrointestinal uptake of sildenafil was confirmed by detecting increased intraocular pressure.**

Intraocular pressure in the sildenafil-treated sheep (CALPSILD, *n* = 6) was significantly higher at 2 and 4 h after oral administration, compared to baseline and compared to the untreated sheep (CONTROL+CALP, *n* = 6 + 5). Time effects: ** *p* < 0.01 vs. 0h. Group effects: ^#^ *p* < 0.05 vs. CONTROL+CALP. Bars are means ± SD.
